# Complete Genome Sequence of two *Gordonia rubripertincta* cluster CS4 Bacteriophages, Vitaenoii and Philon9

**DOI:** 10.17912/micropub.biology.001957

**Published:** 2026-04-07

**Authors:** Joshua T. McMenemy, Amanda I. Acra, Elisabeth A. Alexander, Vanessa K. Cordovez, Morgan M. Fitzpatrick, Dayanara Mejia, Chaeli H. Smith, Marie P. Fogarty

**Affiliations:** 1 Durham Technical Community College, Durham, North Carolina, United States; 2 Science and Math, Durham Technical Community College, Durham, North Carolina, United States

## Abstract

We report the discovery and genome sequences of bacteriophages Vitaenoii and Philon9, isolated from soil samples collected in Durham, North Carolina using the host *Gordonia rubripertincta* NRRL B-16540. The phages have siphoviral morphology and are assigned to bacteriophage subcluster CS4.

**Figure 1. Transmission electron microscopy and comparative genomics analysis of Vitaenoii and Philon9:** Negative stain (2 % phosphotungstic acid, PTA) TEM images of Vitaenoii (A) and Philon9 (B) show siphoviral morphology, with long and flexible tails. Scale bar = 200 nm. C: Heatmap showing the structure of proteomic equivalence quotient (PEQ) among CS4 subcluster phage. Vitaenoii and Philon9 cluster together within the CS4 subcluster (blue box). The PhamClust workflow was used to calculate PEQ, defined as the extent of amino acid sequence similarity among shared genes within the CS4 subcluster, and to generate the resulting heatmap.

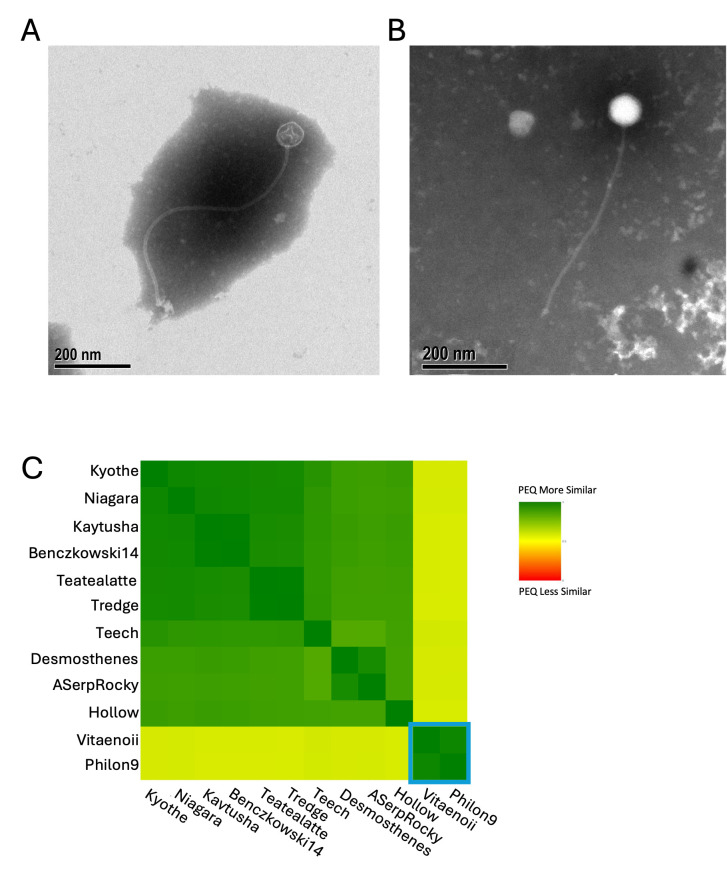



## Description

*Gordonia* are ubiquitous Gram-positive bacteria belonging to the phylum Actinobacteria. *Gordonia* species are found across diverse environments and hold potential for environmental and industrial biotechnological application (Arenskötter et al., 2004, Pope et al., 2017). Exploring the genetic diversity of bacteriophages that infect *Gordonia* species can advance our knowledge of these bacteria (Arenskötter et al., 2004). To contribute to our understanding of *Gordonia* bacteriophage diversity and evolution, we describe the isolation and characterization of two lytic bacteriophages that infect *Gordonia rubripertincta* strain NRRL B-16540, Vitaenoii and Philon9.

Vitaenoii and Philon9 were extracted from soil samples collected in Durham, NC (Table 1). Standard enrichment isolation procedures were used (Zorawik et al., 2024). Briefly, the soil samples were washed with peptone-yeast extract-calcium (PYCa) liquid medium to extract bacteriophages, and the resulting washes were filtered through 0.22 µm filters. The filtrates were inoculated with *G. rubripertincta* NRRL B-16540 and incubated with shaking at 30˚C for 2-5 days. Aliquots of the resulting cultures were filtered, spotted on PYCa top agar containing *G. rubripertincta* and incubated at 30˚C for 3 – 5 days. Vitaenoii and Philon9 produced clear plaques with well-defined borders between 0.25 – 0.5 mm in size (n = 4). Three rounds of individual plaque plating were carried out before lysates were prepared. All plaque assays were incubated for 2–5 days at 30˚C. Lysates were negatively stained using 2 % phosphotungstic acid (PTA) and imaged by transmission electron microscopy (TEM) to reveal siphovirus morphologies with very long tails measuring over 400 nm in length (Table 1, Figure 1).

Genomic DNA was isolated from lysates using phenol-chloroform-isoamyl alcohol extraction (Sigma-Aldrich, P2069). Genome sequencing was performed by the Pittsburgh Bacteriophage Institute, using the NEB Ultra II FS kit for library preparation. For genome sequencing, Illumina NextSeq 1000 (with XLEAP-SBS P1 Kit) was used for Vitaenoii to generate 2,804,678 100 base single-end reads and Illumina MiSeq was used for Philon9, to generate 462,218 150 base single-end reads. For Vitaenoii, raw reads were trimmed with cutadapt 4.7 (using the option: –nextseq-trim 30) and filtered with skewer 0.2.2 (using the options: -q 20 -Q 30 -n -l 50) prior to assembly. &nbsp;Sequence reads were assembled using Newbler v2.9 and genomes were checked for accuracy and completion using Consed v.29 (Silva et al., 2013, Gordon and Green, 2013, Russell, 2018). Both phages have direct terminal repeat genome ends. The terminal repeat lengths are reported in Table 1, along with number of reads, average genome coverage and genome length, and GC content for each phage.&nbsp;Vitaenoii and Philon9 show 99% nucleotide identity as compared to 88% nucleotide identity with Niagara, the next most closely related phage outside the pair.

Gene annotation for each genome was performed using DNA Master v5.23.6 (Pope and Jacobs-Sera, 2018) and PECAAN (Reinhart et al., 2016), platforms integrating multiple bioinformatic gene prediction tools and genomic databases. The genomes were automatically annotated using Glimmer v3.02b (Delcher et al., 2007), and GeneMark v2.5p (Besemer & Borodovsky, 2005) to identify open reading frames and assess coding potential and potential gene start sites. Gene start site refinement and putative gene function assignments were determined using Starterator v1.2 ( http://phages.wustl.edu/ starterator /), Phamerator (Actino_draft database v578 (Cresawn et al., 2011)), BLASTP searches against NCBI non-redundant and Actinobacteriophage databases (Altschul et al., 1990), and HHpred &nbsp;searches against the PDB_mmCIF70, Pfam-A_v36, UniProt-SwissProt-viral70_3, NCBI_Conserved_ Domains(CD)_v3.19 databases (Zimmermann et al., 2018). Deep TMHMM v1.0.24 was used to detect putative transmembrane domains (Hallgren et al., 2022), and Aragorn v1.2.41 (Laslett and Canback, 2004) and tRNAscan-SE v2.0 (Lowe and Chan, 2016) were used for tRNA prediction. Default settings and parameters were used for all software, unless otherwise specified. The GC content of the Vitaenoii genome is 58.8%, and the Philon9 genome is 58.7%. Based on gene content similarity (GCS) of at least 35% to phages in the Actinobacteriophages database, phagesDB (Russell and Hatfull, 2017 , Pope et al., 2017), Vitaenoii and Philon9 were assigned to bacteriophage cluster CS, and subcluster CS4. When compared to the additional 10 members of the CS4 subcluster, Vitaenoii and Philon9 show notable similarity to each other based on nucleotide sequence similarity, GCS and PhamClust analysis ( Figure 1C and Table 1). PhamClust is a computational approach that efficiently calculates a proteomic equivalence quotient (PEQ) value for each bacteriophage pair based on amino acid sequence identity of shared genes, enabling accurate clustering and subclustering. The resulting heatmap generated allows visualization of cluster structure, subclusters and potential outliers (Gauthier & Hatfull, 2023). As with other cluster CS4 bacteriophage, genes involved in structure and assembly are in the first third of the left arm of the genome and encoded on the forward strand. Genes involved in DNA metabolism (including CobT-like cobalamin biosynthesis protein, Cas4 family exonuclease, nucleoside deoxyribosyltransferase, and ASCE-ATPase) and replication (including DNA primase, DNA helicase, DNA polymerase III sliding clamp (beta) and DnaQ-like DNA Polymerase III subunit) are scattered among the rest of the genome and encoded on the reverse strand. As with previously characterized cluster CS bacteriophages, no integrase or immunity repressor functions could be identified, suggesting they are unlikely to establish lysogeny and consistent with our inability to raise lysogens using standard procedures.&nbsp;

**&nbsp;Nucleotide sequence accession numbers**

Vitaenoii is available at GenBank with Accession No. PV915899 and Sequence Read Archive No. SRX28943186 . Philon9 is available GenBank with Accession No. PX089653 and Sequence Read Archive No. SRX28943174 .

Table 1: Isolation and sequencing parameters, and phage characteristics

**Table:** 

Bacteriophage	Vitaenoii	Philon9
GPS coordinates	36.075457 N, 79.007587 W (Eno River State Park Durham, NC 27705)	36.061757 N, 78.91479 W (Yard of home in Durham, NC 27704)
Plaque size (mm)	0.25-0.5mm (n = 4)	0.25-0.5mm (n = 4)
Capsid size (nm) &nbsp;	65.9± 5.9nm &nbsp;(n=7)	69.1± 7.9nm (n=4)
Tail length (nm)	489.0 ± 74nm (n = 7)	479.3 ± 5nm (n = 4)
Number of Reads	2,804,678	462,218
Average Fold Coverage	2804	73
Genome Length (bp)	74721	74736 &nbsp;
Genome End Direct Terminal Repeat	1206 bp	1205 bp
GC content %	58.8%	58.7%
Nucleotide similarity	99%	99%
Gene Content Similarity (GCS)	100 % &nbsp;	100 % &nbsp;
Number of genes with predicted function / Total predicted genes	39/96	39/96
